# Cell sorting microbeads as novel contrast agent for magnetic resonance imaging

**DOI:** 10.1038/s41598-022-21762-8

**Published:** 2022-10-21

**Authors:** Aman Khurana, Francesc Marti, David K. Powell, J. Anthony Brandon, Adam Dugan, Roberto Gedaly, Fanny Chapelin

**Affiliations:** 1 Department of Radiology, University of Kentucky, Lexington, KY USA; 2 F. Joseph Halcomb III, M.D. Department of Biomedical Engineering, University of Kentucky, 514F RMB, 143 Graham Avenue, Lexington, KY 40506-0108 USA; 3 Lucille Parker Markey Cancer Center, University of Kentucky, Lexington, KY USA; 4 Transplant Division, Department of Surgery, University of Kentucky, Lexington, KY USA; 5 Department of Neuroscience, University of Kentucky, Lexington, KY USA; 6 Sanders Brown Center on Aging, University of Kentucky, Lexington, KY USA; 7 Department of Biostatistics, University of Kentucky, Lexington, KY USA

**Keywords:** Imaging, Magnetic resonance imaging, Imaging the immune system, Transplant immunology

## Abstract

The success of several cell-based therapies and prevalent use of magnetic resonance imaging (MRI) in the clinic has fueled the development of contrast agents for specific cell tracking applications. Safe and efficient labeling of non-phagocytic cell types such as T cells nonetheless remains challenging. We developed a one-stop shop approach where the T cell sorting agent also labels the cells which can subsequently be depicted using non-invasive MRI. We compared the MR signal effects of magnetic-assisted cell sorting microbeads (CD25) to the current preclinical gold standard, ferumoxytol. We investigated in vitro labeling efficiency of regulatory T cells (Tregs) with MRI and histopathologic confirmation. Thereafter, Tregs and T cells were labeled with CD25 microbeads in vitro and delivered via intravenous injection. Liver MRIs pre- and 24 h post-injection were performed to determine in vivo tracking feasibility. We show that CD25 microbeads exhibit T2 signal decay properties similar to other iron oxide contrast agents. CD25 microbeads are readily internalized by Tregs and can be detected by non-invasive MRI with dose dependent T2 signal suppression. Systemically injected labeled Tregs can be detected in the liver 24 h post-injection, contrary to T cell control. Our CD25 microbead-based labeling method is an effective tool for Treg tagging, yielding detectable MR signal change in cell phantoms and in vivo. This novel cellular tracking method will be key in tracking the fate of Tregs in inflammatory pathologies and solid organ transplantation.

## Introduction

Rapid evolution and prevalent use of magnetic resonance imaging (MRI) in the clinic has fueled research in the development of contrast agents and methods to improve pathological tissue delineation and to visualize specific cell trafficking in vivo. The first reported use of contrast agents in human MRI studies employed oral ferric chloride to monitor bowel metabolic processes^1^. Nowadays, 25–40% of all clinical MRIs employ contrast agents for tissue enhancement^2^ and numerous clinical studies have proven the feasibility of using MRI for longitudinal tracking of specific cell populations^3^.

MRI signal intensity and contrast is controlled by numerous intrinsic factors, including spin density, tissue susceptibility, T1 and T2 relaxation times and physiological motion^4^. Extrinsic contrast agents usually target proton relaxation. They are composed of paramagnetic gadolinium^5^, manganese^6^ ion complexes or superparamagnetic iron oxide particles nanoparticles (SPIO)^7^. Although gadolinium chelates are currently the most common clinically available option, lack of labeling specificity as well as gadolinium tissue accumulation^8^ and freshwater contamination^9^ concerns have rekindled interest for alternative agents. Accumulation of superparamagnetic iron oxides (SPIOs) in tissue provides excellent contrast by altering the T1 and T2 relaxation times of adjacent protons, which aids in differentiating these tissues from the adjacent normal tissue signals^10^.

Ferumoxytol (Feraheme) is an FDA approved iron-rich nanoparticle originally designed for intravenous treatment of patients with iron deficiency^11–13^. Following uncovering of its MR properties, ferumoxytol has been extensively used off-label for cell tracking in numerous applications, such as cancer immune microenvironment, immune disorders, and cell transplant imaging in preclinical studies^14–16^. Cell types presenting with low phagocytic activity, such as T cells, are not easily labeled with existing contrast agents like ferumoxytol. Additionally, in vitro manipulations to increase cell labeling also exacerbates risks of cell death or cell transformation^17,18^. Reducing in vitro handling also directly affects time and sterility concerns when using regulatory T cells (Tregs) in a transplant surgery setting.

Magnetic-activated cell sorting (MACS) is a common method for cell population separation from tissue homogenates^19^. The technique relies on targeting cell surface markers with specific antibodies bound to superparamagnetic nanoparticles also called ‘microbeads’. When placed in a high gradient magnetic field, the labeled cells remain trapped while unlabeled cells elute. Labeled cells can be collected by removing the magnetic field. Our hypothesis is that the microbeads used for cell separation have magnetic properties pertinent to cell labeling and tracking applications. In this study, we aim to compare the MR signal effects of MACS microbeads (CD25) to the preclinical off label gold standard ferumoxytol and explore feasibility of in vivo CD25-labeled Treg tracking.

## Results

### Microbeads MRI characteristics

**Figure 1:**
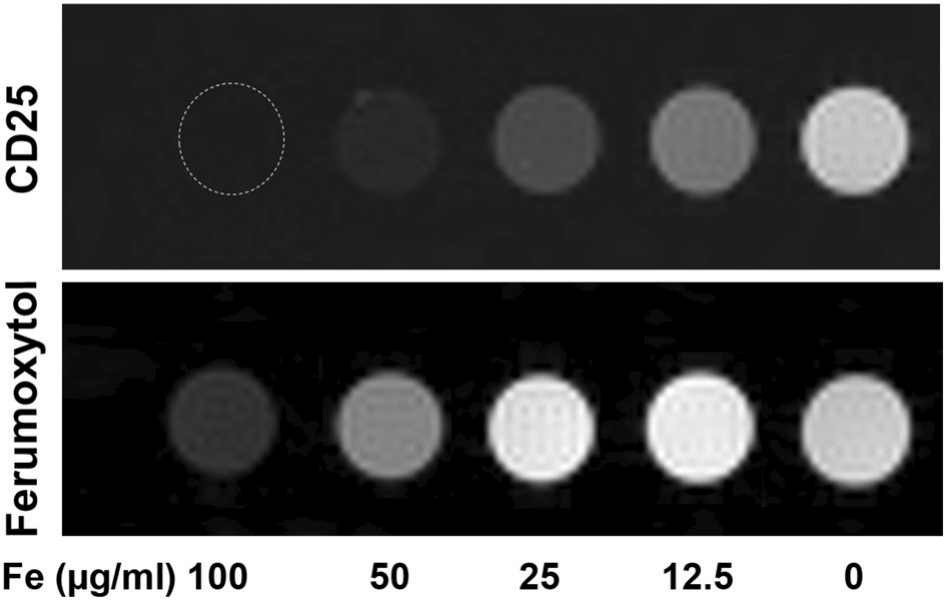
Magnetic resonance signal effects of CD25 microbeads compared to ferumoxytol. T2-weighted magnetic resonance images of agarose gels (200 µl) containing increasing contrast agent concentrations (0, 12.5, 25, 50 or 100 µg/ml of CD25 microbeads or ferumoxytol) show marked signal decrease (dark) for both contrast agents compared to controls (0 = agarose gel alone). Signal decay is more pronounced for CD25 microbeads compared to ferumoxytol. Data was acquired on a 7 T MRI with 10 TEs equally spaced from 6.4 to 64 ms.

**Figure 2:**
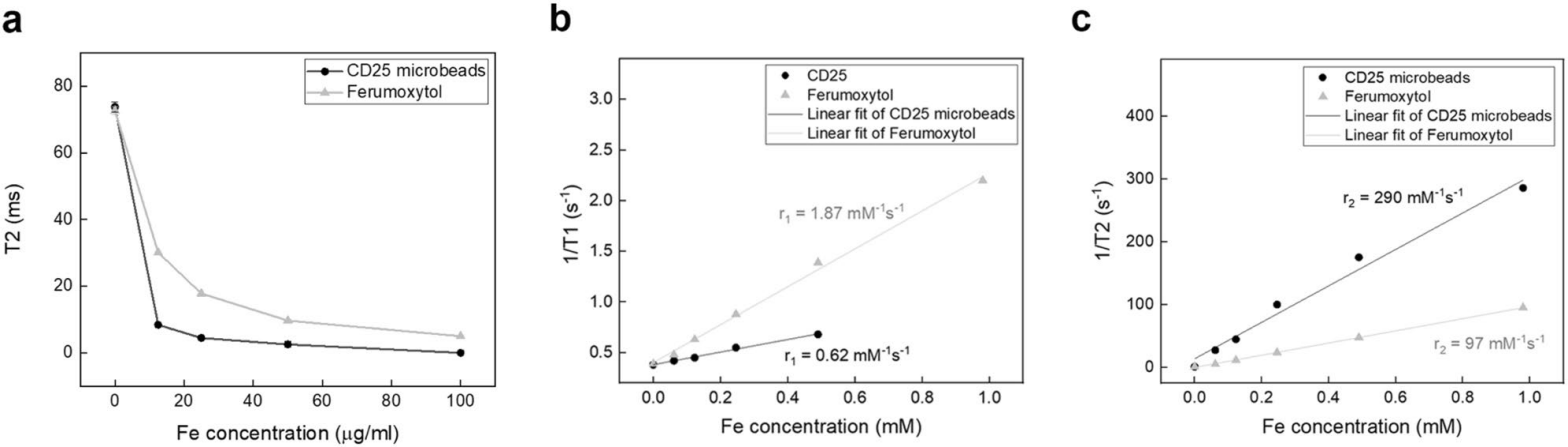
Quantitative T2 decay as a function of iron concentration and relaxivity measurement. (**a**) As expected, T2 values for CD25 microbeads and ferumoxytol follow an decreasing curve with increasing iron amount. T2 decay is more pronounced in CD25 microbead samples compared to ferumoxytol samples. Data are presented as ROI mean ± standard deviation. (**b**) The inverse of T1 values were plotted against iron concentration to yield CD25 microbead and ferumoxytol r_1_. (**c**) The inverse of T2 values were plotted against iron concentration to yield CD25 microbead and ferumoxytol r_2_. CD25 microbeads display much larger r_2_/r_1_ ratio than ferumoxytol (r_2_/r_1_ = 468 and 52 respectively).

We tested the magnetic properties of CD25 microbeads compared to cell tracking standard ferumoxytol (Fig. 1) and agarose gel controls. Both microbeads exhibit increased T2 shortening with increasing concentration, with ferumoxytol demonstrating a weaker effect compared to CD25 microbeads (Fig. 1). As expected, T2 times decrease in relation to CD25, and ferumoxytol concentration (Fig. 2a). T2 decay is more pronounced in CD25 microbead samples compared to ferumoxytol samples. The inverse of T1 and T2 values were plotted against iron concentration and the linear fit yielded CD25 microbead and ferumoxytol relaxivities r_1_ and r_2_ (Fit 0.983 < R^2^ < 0.999). The measured relaxivities of ferumoxytol at 7 T are r_1_ = 1.87 mM^−1^ s^−1^ and r_2_ = 97 mM^−1^ s^−1^; r_2_/r_1_ = 52 , and those of CD25 microbeads are r_1_ = 0.62 mM^−1^ s^−1^ and r_2_ = 290 mM^−1^ s^−1^; r_2_/r_1_ = 468 (Fig. 2b,c). The r_2_ relaxivity difference is consistent with particle size, as CD25 microbeads have a larger iron core compared to ferumoxytol^20^.

**Figure 3:**
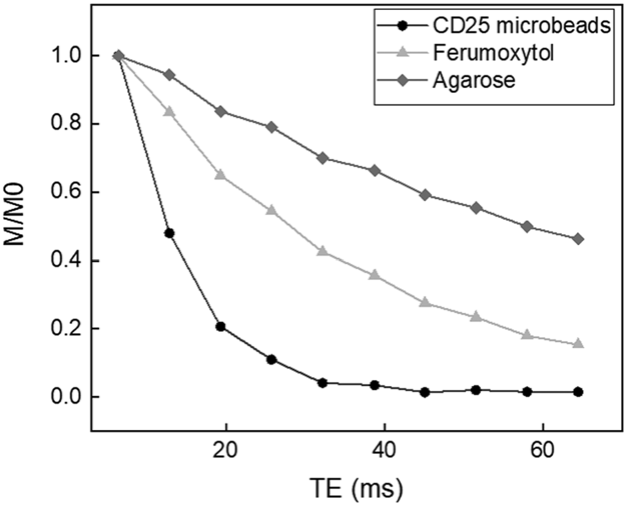
T2 relaxation curves of microbeads. CD25 microbeads (12.5 µgFe/ml) and ferumoxytol (12.5 µgFe/ml) display shortened T2 relaxation compared to agarose gel which exhibits linear signal decay with respect to echo time.

Regarding T2 relaxation curves, ferumoxytol exhibits moderate T2 effects compared to agarose which exhibits linear relaxation within the echo time range selected (Fig. 3), indicating somewhat long inherent T2 and modest impact on MRI signal. However, at equivalent iron concentrations, CD25 microbeads display comparably sharp responses (Fig. 3), demonstrating CD25 microbeads’ ability to generate strong T2 signal decay on MR images.

### Cell internalization of the microbeads

**Figure 4:**
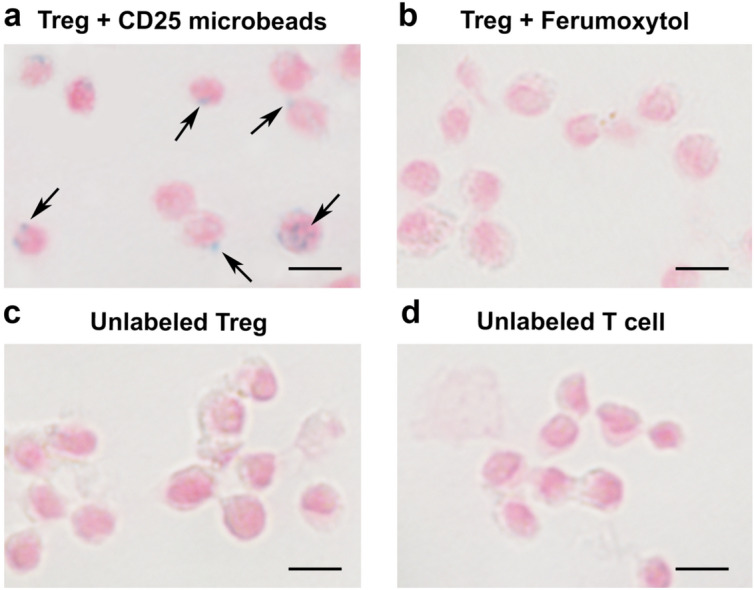
Confirmation of CD25 microbead intracellular uptake by Tregs. Regulatory T cells labeled overnight with 10 μl CD25 microbeads (45 μgFe/ml) exhibit positive intracellular Prussian blue staining (**a,** arrows). Tregs labeled with ferumoxytol overnight (**b**) do not internalize the contrast agent. Unlabeled control Treg (**c**) and T cells (**d**) do not exhibit staining.

Following MACS sorting, CD25 positive Tregs account for approximately 15% of all isolated T cells. Overnight Treg cell labeling with CD25 microbeads shows positive intracellular Prussian Blue staining, indicating successful uptake of CD25 microbeads (Fig. 4a) without viability impairment (94% viable cells, *p* < 0.01 compared to unlabeled controls). CD25-labeled T cells and unlabeled controls do not exhibit positive staining (Fig. 4c,d), signifying that CD25 needs to be present at the surface of the cells to enable internalization of the microbead. Intracellular labeling with ferumoxytol is ineffective (Fig. 4b), likely due to the non-phagocytic nature of T cells. These findings reinforce the potential of using CD25 microbeads as a cell tracking agent.

**Figure 5:**
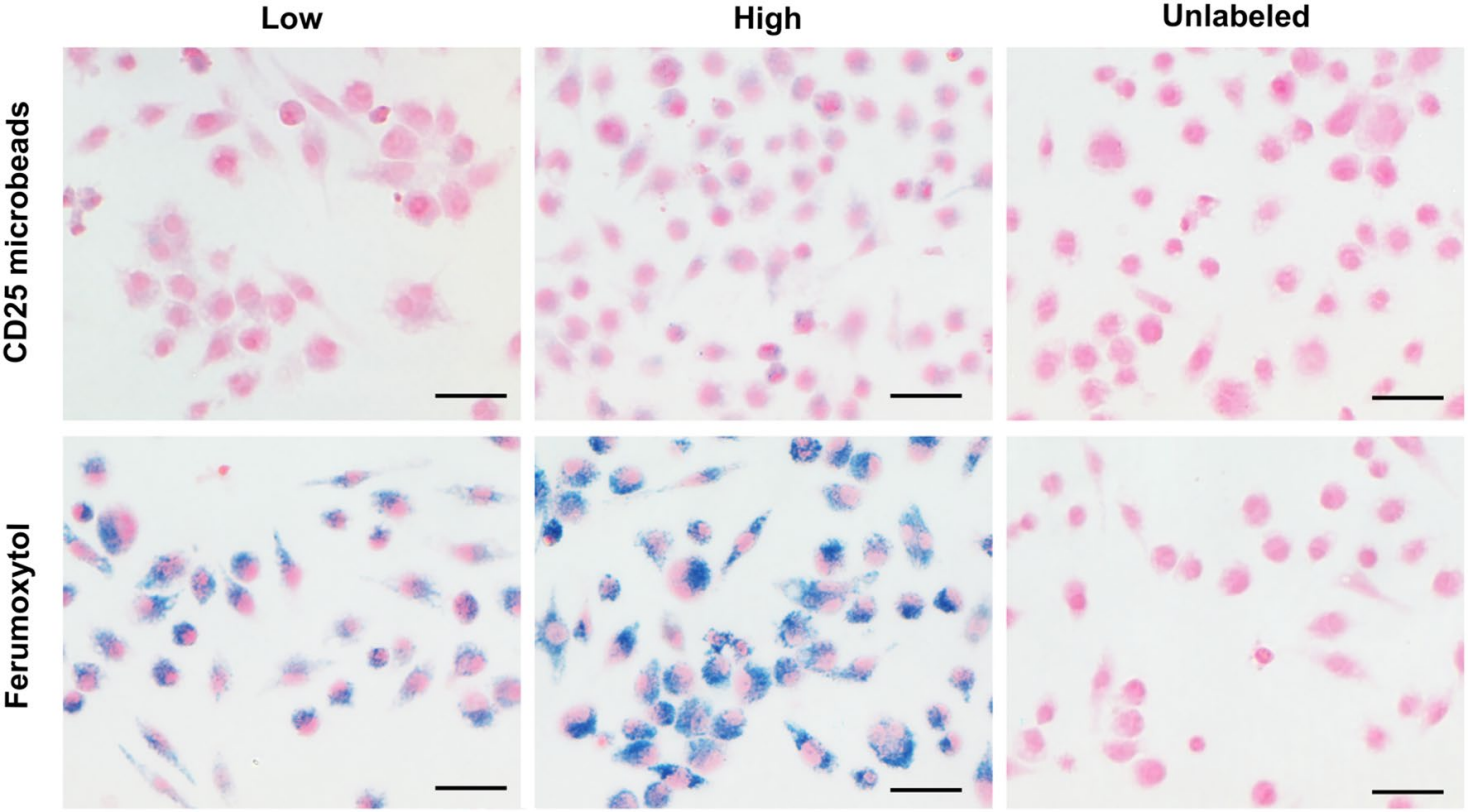
Histological assessment of nanoparticle uptake by macrophages. Light microscopy images of macrophages labeled with varying concentrations of CD25 microbeads (top row, low = 10 and high = 20 µl CD25 microbeads respectively) or ferumoxytol (bottom row, low = 200 and high = 400 µgFe/ml respectively) are displayed. Data shows strong intracellular localization of ferumoxytol (blue)**,** conversely to CD25 microbeads, suggesting that the CD25 antibody does not support internalization of the beads by unspecific phagocytic cells. Scale bar represents 100 µm.

To confirm CD25 microbead specificity towards regulatory T cells, we incubated murine macrophages, an unspecific phagocytic cell type, with either ferumoxytol or CD25 microbeads (Fig. 5). In each case, the findings are reversed compared to Tregs. As expected and previously reported, ferumoxytol is abundantly phagocytosed by macrophages (Fig. 5) as evidenced by the ample intracellular Prussian Blue deposits. CD25 microbeads-labeled macrophages, on the other hand, exhibit minimal positive staining (Fig. 5).

### MRI of CD25 microbead-labeled Treg phantoms

**Figure 6:**
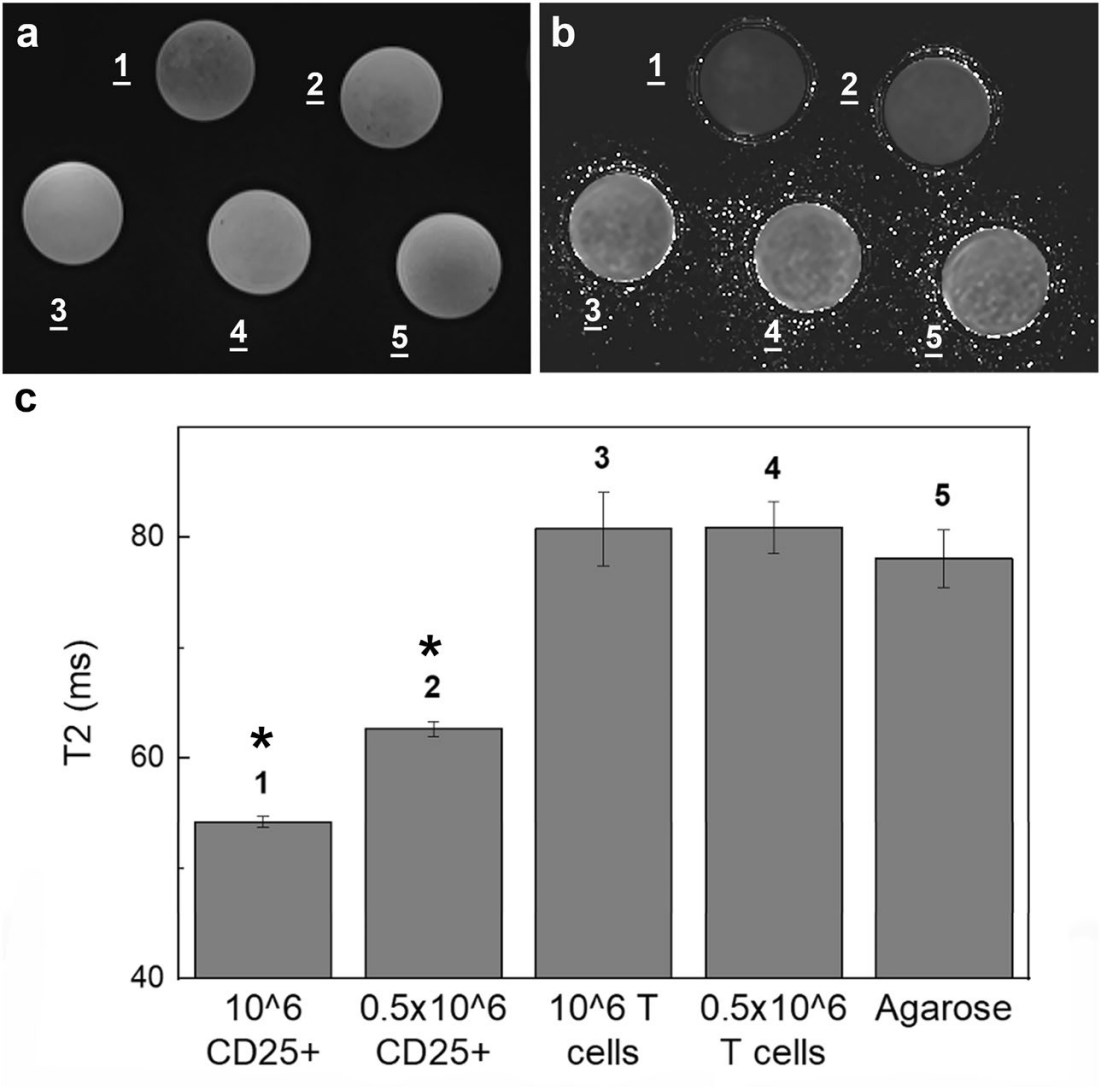
Magnetic resonance signal effects of CD25 microbead-labeled Tregs. T2-weighted magnetic resonance images (**a**) and corresponding T2 relaxation time maps (**b**) of agarose gels containing CD25 microbead-labeled Tregs (1–2) show marked signal reduction compared to unlabeled T cells (3–4) and agarose (5) controls. Quantitative analysis of the T2 relaxation times (**c**) confirms significant T2 shortening in CD25-microbead labeled cells. T2 of half million Tregs is 22% lower than the T2 of unlabeled T cells and agarose alone (*P = 0.0001 and 0.0006 respectively). T2 is further shortened (33%) in one million labeled Treg samples (*P = 0.001 compared to half million Treg sample). Data are presented as mean ± standard deviation.

CD25 microbead-labeled Tregs (Fig. 6a, 1–2) displayed decreased signal on T2-weighted MR images compared to unlabeled cells (Fig. 6a, 3–4) and agarose controls (Fig. 6a, 5), consistent with adequate labeling with a paramagnetic iron oxide. Corresponding T2 relaxation maps (Fig. 6b) yielded quantitative T2 signal decay. The T2 of half million Tregs (T2_(2)_ = 62.67 ms ± 0.67) is significantly lower than the T2 of unlabeled T cells (T2_(4)_ = 80.89 ms ± 2.34) and agarose alone (T2_(5)_ = 78.11 ms ± 2.59, *P* = 0.0001 and 0.0006 respectively, Fig. 6c). T2 signal is further shortened in one million labeled Treg samples (T2_(1)_ = 54.22 ms ± 0.51) and significantly lower than the half million labeled Treg samples (*P* = 0.0001, Fig. 6c).

### In vivo MRI of CD25 microbead-labeled Treg

**Figure 7:**
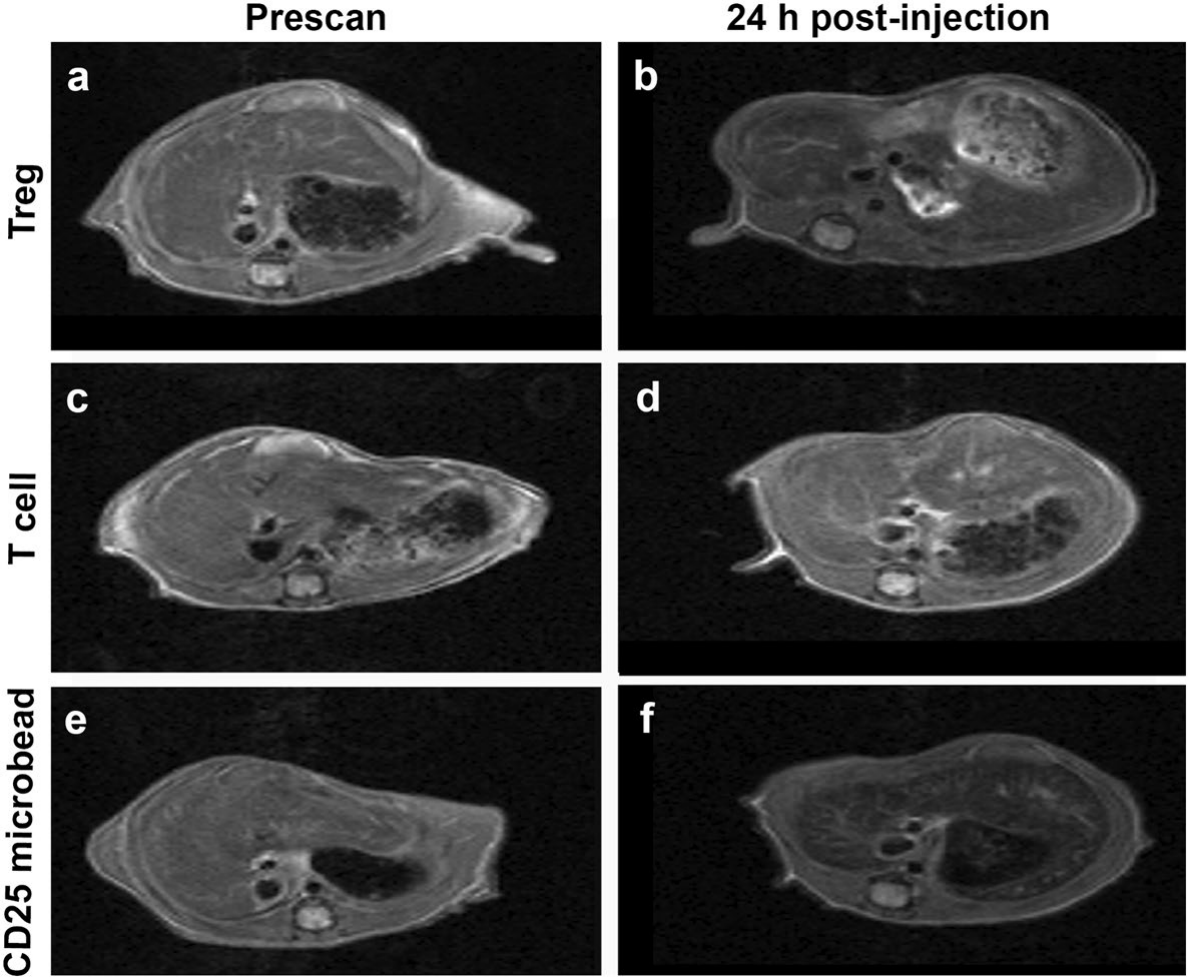
Axial T2-weighted liver MR images of mice pre and 24 h post-intravenous injection of labeled cells or CD25 microbeads. Representative images of CD25 microbead-labeled Treg receiving mouse pre (**a**) and 24 h-post (**b**) IV injection show T2 signal reduction compared to CD25 microbead-labeled T cells (**c** and **d**, negative control). Positive control mice receiving CD25 microbeads alone intravenously show marked T2 signal decrease 24 h post-injection (**e,f**).

**Figure 8:**
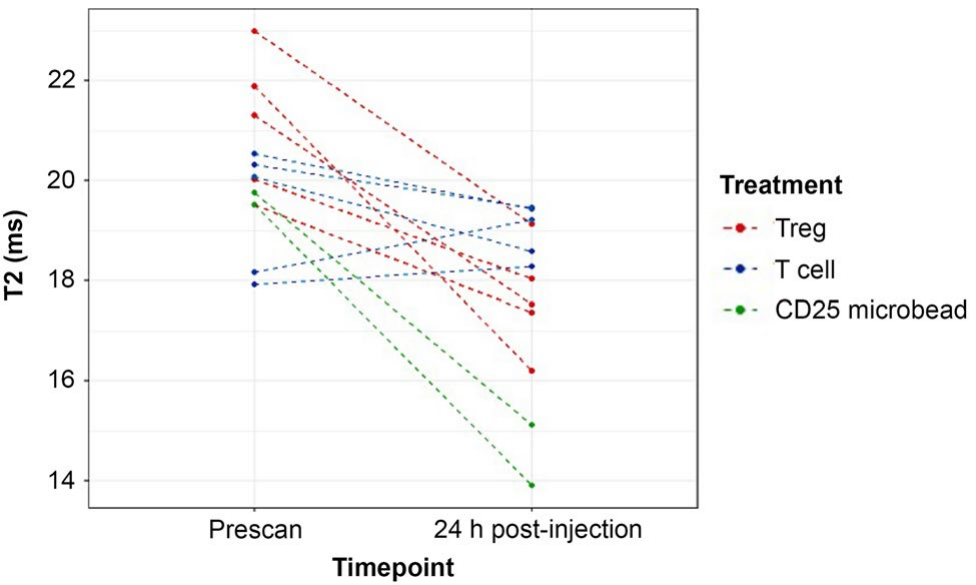
Quantitative summary of in vivo T2 measurements. T2 relaxation times show significantly shorter T2 values in Treg-receiving mice 24 h post-injection compared to prescan (*p* = 0.007). Contrarily, T cell-receiving mice show no differences in T2 (*p* = 0.44). Positive control mice receiving CD25 microbeads alone show drastic T2 reduction as expected. The difference between Treg and T cell receiving mice is also significant (*p* = 0.029). Data are displayed as T2 means for each animal (9 measurements per animal per data point).

Mice receiving CD25 microbead-labeled Tregs exhibited decreased liver signal on T2-weighted MR images 24 h after systemic injection (Fig. 7a,b) compared to negative control mice receiving CD25 microbead-labeled T cells (Fig. 7c,d). Mice receiving CD25 microbeads alone systemically also displayed strong T2 signal decay at 24 h post-injection (Fig. 7e,f). Quantitative analysis of T2 values in mice receiving CD25 microbead-labeled Tregs showed significant T2 change between pre and 24 h post MRI (*p* = 0.007, Fig. 8). This was not the case in CD25 microbead-labeled T cells (*p* = 0.44, Fig. 8). ANOVA comparing T2 change in all mice confirmed significant signal change in mice receiving Treg compared to T cells and microbeads (*p* = 0.029 and *p* = 0.007 respectively, Fig. 8). These results confirm the feasibility of Treg tracking in vivo.

## Discussion

This study demonstrates that the CD25 microbeads used for magnetic sorting of regulatory T cells constitute an excellent cell labeling agent for subsequent MR tracking. The specific antibody binding promotes internalization of the magnetic bead, leading to strong MR effects, which have so far been unattainable with other SPIOs. To our knowledge, successful labeling of T cells with clinically applicable agents like the gold standard ferumoxytol or the CD25 microbeads described here has not been reported in the literature.

The CD25 microbeads that serve as MRI contrast agents in this project are approved for clinical research (NCT00675831, NCT03284242). CD25 microbeads alone displayed strong MR T2 effect, superior to the standard ferumoxytol, which is also used ‘off label’ for cell tracking applications. Equivalent CD34 microbeads are FDA approved as a humanitarian use device for treatment of patients with acute myeloid leukemia^21,22^, which should fast-track approval for clinical ‘off-label’ use of CD25 microbeads, thereby enabling researchers and clinicians to track regulatory T cells in vivo. In addition, we confirmed that CD25 microbeads generate significant contrast on a 1.5 T clinical scanner (data not shown) and sequence adjustments can be made to recover sensitivity loss when moving to lower magnetic fields.

Due to their low cytoplasmic capacity and phagocytic activity, T cells have consistently been challenging to label for MR tracking applications^23,24^. Bulte et al. were the first team to label T cells with a similar magnetic sorting bead strategy, but that technique required a biotin-streptavin intermediate and was not pursued for in vivo tracking^25^. Yeh et al. were able to label T cells with iron oxide particles but the specific contrast agents used have since been discontinued^26^. Jin et al. reported successful labeling of T cells using Molday Ion, a preclinical ultrasmall SPIO, in a murine stroke model^27^. Liu et al. developed pegylated iron nanoparticles that were internalized in Jurkat cells and detected the infused cells by MRI in a heart transplant rejection model^28^. Chandrasekharan et al. used similar Miltenyi microbeads to label neutrophils for subsequent tracking with magnetic particle imaging, which is to date not clinically available^29^. Gordon-Wylie et al. also confirmed the potential of antibody conjugated nanoparticles (NP) for imaging^30^, but these NP remain in the early stages of development, whereas the CD25 microbeads used here are already part of the autologous Treg isolation workflow in the clinic. Garden et al. ornated their ultrasmall SPIO with TAT peptides to facilitate labeling, yielding both intracytoplasmic and intranuclear labeling^31^. Adding contrast agents and transfection reagents to improve cell labeling presents a major limitation for subsequent FDA approval as adding external agents increases the risk of introducing pathogens and cellular atypia^17,18,32^.

When used for labeling Treg cells, CD25 microbeads were able to initiate internalization, likely through specific binding to the CD25-containing high affinity IL2 receptor^33^. The endocytic mechanism is probably the result of a clathrin-independent, cargo-induced internalization of the IL2 receptor^34,35^. T cells that do not express CD25 were not able to bind and internalize the CD25 microbeads (data not shown). We were able to detect a significant MR signal change with as few as 0.5 × 10^6^ cells in a large voxel. Conversely, ferumoxytol was not internalized in Tregs. Using the microbeads which are already used for Treg cell sorting can therefore obviate unnecessary cell manipulation with acceptable labeling efficiency. Our labeling method would provide one-stop shop approach where the Treg sorting step also labels the cells which can be readily tracked once they home to specific organs using non-invasive MRI.

While CD25 microbeads specifically labeled T regs, they were not as readily internalized by macrophages. Macrophages and other phagocytes such as dendritic cells have been an MR cell labeling reference for decades for both practical and clinical reasons^15,36–41^. Due to their phagocytic nature, uptake of large amounts of contrast agents is comparatively straightforward^36,38,42,43^ thereby enabling elegant non-invasive investigation of immune processes in vivo in cancer, auto-immune and transplant applications^39,40,44–47^. Although peculiar^48,49^, the finding that CD25 microbeads are not as easily internalized by macrophages offers a desirable edge over the gold standard ferumoxytol (Fig. 5) for in vivo applications. Uptake of free CD25 contrast from dead Tregs by macrophages resulting in confounding MR signal may therefore be minimized in vivo*.* Qie et al. showed that surface modification of nanoparticles enables evasion from phagocytosis in certain applications^50^. Based on this, our hypothesis is that the CD25 antibodies block access to the dextran coating, thereby altering the phagocytic activity towards the microbeads.

T2 effects of intracellular iron oxide-based contrast agents (ferumoxytol & CD25 microbeads) can be measured in the tissues where therapeutic cells distribute via creation of T2 maps. Imaging-based cellular tracking is reliable, reproducible, and non-invasive with MRI^14,51,52^. Multiple animal and clinical MRI scanners have in-built multi-echo sequences with T2 map calculation capability to quantify dynamic change of hypointense clusters in the organ of the interest^27^. One limitation of iron oxide contrast agents is that it only enables semi-quantitative assessment of cell infiltration in a given tissue rather than an absolute number. Fluorinated contrasts agents have shown promise for the labeling and fully quantitative tracking of T cells^53–57^. Nonetheless, this method requires fluorine-specific MRI hardware and software that is only sparsely available in the clinical setting to date^16^.

Transplant graft rejection is driven by a decline in the frequency of Treg cells or impairment in Treg cell function, which controls alloantigen specific T cells^58,59^. To prevent graft rejection, shifting the immune balance via adoptive transfer of Treg cells has become a very promising therapeutic option^60,61^. Oo et al. have demonstrated that approximately 22–44% of infused Tregs homed to and were retained in the livers of patients with autoimmune hepatitis for up to 72 h after leukapheresis, labeling, and re-infusion^62^. The authors used indium isotope for labeling and tracked the cells with single photon emission computed tomography (SPECT-CT), which utilizes ionizing radiation. To date, there are no MRI based cellular tracking methods for Tregs. More importantly, success of Treg homing to the transplant may be an early marker of organ-targeted efficacy and transplant acceptance and should be determined non-invasively. Our platform which integrates the CD25 sorting agent as an ‘off label’ contrast agent may become a key resource in determining homing and fate of Tregs in inflammatory pathologies and solid organ transplantation.

In conclusion, our novel CD25 microbead-based labeling method is an effective tool for Treg tagging, yielding detectable MR signal change in vivo. Further experiments are warranted to unlock the full translational potential of this novel cellular labeling method. Nonetheless, given use of this agent in the existing clinical Treg sorting workflow, this method offers ease of use for Treg labeling during transplant surgery given time and sterility constraints.

## Materials and methods

### Contrast agents

Microbeads developed by Miltenyi Biotec for magnetic-assisted cell sorting (MACS) are composed of an antibody tethered to a dextran-coated iron bead. Here, we used the CD25 microbeads (CliniMACS CD25, Miltenyi Biotec, Somerville, MA) which enable positive regulatory T cell (Treg) sorting. The beads average 50 nm in diameter^63^. The iron concentration of CD25 microbeads was determined by acid dissolution and established UV–VIS spectrophotometric method^25,64^. CD25 microbeads were measured at 2.24 mgFe/ml.

Ferumoxytol (AMAG Pharmaceuticals, Waltham, MA) nanoparticles are polymer-coated iron cores with a mean diameter of 30 nm^65^. Ferumoxytol’s magnetic properties and its use for cell labeling and subsequent tracking are well studied^47,66–70^.

### Bead phantom preparation

Phantoms were prepared by diluting different amounts of CD25 microbeads or ferumoxytol in 1% agarose for a final volume of 200 µl. Concentrations tested included 0, 12.5, 25, 50 and 100 µgFe/ml. Dilutions were chosen based on in vitro working volumes that would be suitable for preserved cell viability.

For relaxivities measurements, phantoms were prepared by diluting different amounts of CD25 microbeads or ferumoxytol in PBS for a final volume of 200 µl. Concentrations tested included 0, 3.1, 6.25, 12.5, 25 and 50 µgFe/ml. Dilutions were chosen to obtain optimal recovery curves for T1 and T2 measurement at different concentrations.

### MRI measurements of bead phantoms

All MRI images in this study were acquired on a preclinical Bruker BioSpec 7 T imaging system (Bruker, Billerica, MA) using a mouse birdcage body coil (Bruker). Axial images of phantoms were acquired using a Turbo RARE sequence with parameters TR/TE = 2000/20 ms, Matrix size = 96 × 96, FOV = 36 × 36 mm^2^, Slice thickness = 1.2 mm, Averages = 2, Slice number = 16. A T2map Multi Slice Multi Echo (MSME) sequence with TR = 2000 ms and 10 TEs equally spaced from 6.4 to 64 ms, Matrix size = 96 × 96, FOV = 36 × 36 mm^2^, Slice thickness = 1.2 mm, Averages = 3, Slice number = 16 was acquired for T2 and r_2_ calculations. A T1map RARE sequence with TE = 25.7 ms and 8 TRs equally spaced from 900 to 5000 ms, Matrix size = 256 × 192, FOV = 36 × 26 mm^2^, Slice thickness = 1.2 mm, Averages = 1, Slice number = 16 was acquired for r_1_ calculations. T1 and T2 values were extracted using the system’s inbuilt Image Sequence Analysis (ISA T2vtr and T1sat, Paravision 360, V3.0) software by defining three circular ROIs in the center of the PCR tubes.

### Regulatory T cell isolation

The study was approved by University of Kentucky’s Institutional Review Board (IRB #48583). Peripheral blood mononuclear cells were isolated from buffy coats of healthy anonymous blood donors (Kentucky Blood Center, Lexington, KY) in accordance with relevant guidelines and regulations via Ficoll density gradient (Histopaque 1077, Sigma Aldrich, St Louis, MO). Informed consent was obtained from all participants. T cells were isolated via EasySep Human T cell Isolation Kit (Stemcell Technologies, Cambridge, MA), and CD25 Microbeads II (Miltenyi Biotec) were used to further isolate CD25^+^ T cells (here referred to as Tregs to model future Treg isolation). Treg and T cells (CD25 negative) were maintained in RPMI media (Corning, Corning, NY) supplemented with 10% fetal bovine serum (Hyclone, GE Healthcare, Marlborough, MA), 1% penicillin streptomycin (VWR, Radnor, PA), and 100 units/ml of recombinant human interleukin 2 (IL-2, Peprotech, Rocky Hill, NJ). On the first day of culture, cells were activated with T cell Transact (Miltenyi Biotec) per manufacturer’s instructions. Cells were expanded for up to 14 days to obtain cell amounts needed.

### Regulatory T cell labeling and histological staining

To optimize labeling efficacy, Tregs and T cells (CD25-) were plated at a density of 2 million cells per ml of full media in 24 well plates in triplicates. CD25 microbeads were added to the culture media in varying amounts (0, 10, 20 µl per million cells, equivalent to 0, 45 and 90 µgFe/ml) and several incubation times (0, 6, 16 h) were tested. Triplicates of Tregs labeled with ferumoxytol with final concentration of 400 µgFe/ml were also prepared. Triplicates of unlabeled Tregs and T cells were also prepared as controls. After incubation, excess label was removed by three washes followed by centrifugation. T cells were resuspended at a density of 1 million cells in 100 µl phosphate buffered saline and gently spread on a histology slide (Superfrost slide, Fisherbrand, Waltham, MA). Samples were allowed to air dry and were subsequently fixed with 10% formalin (VWR, Radnor, PA) for 15 min. Samples were then stained using a Prussian blue iron staining kit (Polysciences, Warrington, PA) consisting of 15-min incubation of 1:1 potassium ferrocyanide and hydrochloric acid followed by 5 min of nuclear fast red counterstain. Samples were thoroughly washed and allowed to fully air dry. Coverslips were secured onto the slide with Permount (Fisher Scientific, Waltham, PA) mounting media. Histological images were acquired on a Nikon Ti-U Microscope (Nikon, Tokyo, Japan) with 20X and 60X objectives.

### Macrophage labeling and histological staining

TIB-67 murine macrophage cells were kindly provided by Melissa Hollifield (Scientist, University of Kentucky) and cultured in DMEM media supplemented with 10% fetal bovine serum and 1% penicillin streptomycin. Triplicate samples of 40,000 cells were plated in chamber slides at a density of 40,000 cells/cm^2^. ferumoxytol was added to each well to reach a final concentration of 200 (low) or 400 (high) µgFe/ml and one well did not receive ferumoxytol and served as control. Alternatively, 10 (low) or 20 (high) µl of CD25 microbeads were added to each well and one beadless well served as control. Chamber slides were kept in the incubator at 37 °C and 5% CO_2_ overnight. Excess contrast agent was aspirated with the media and chamber slides were washed three times before proceeding to histological staining. Samples were fixed and stained for iron and imaged as described above.

### MRI measurement of regulatory T cell phantoms labeled with CD25 beads

Triplicate samples of half and one million Treg were labeled overnight (16 h) with 10 µl CD25 microbeads per million Tregs. After incubation, excess microbeads were removed by three washes followed by centrifugation. Triplicate samples of unlabeled half and one million T cells were also prepared. All cell samples were resuspended in 100 µl agarose gel (1% dilution in PBS) and transferred to 0.2 ml PCR tubes for MR imaging.

MRI images were acquired on the same system as above (Bruker BioSpec 7 T) using a mouse birdcage body coil. Axial images of the cell samples were acquired using a Turbo RARE sequence with parameters TR/TE = 2500/33 ms, Matrix size = 128 × 128, FOV = 28 × 28 mm^2^, Slice thickness = 0.7 mm, Averages = 2, Slice number = 16 and a T2map Multi Slice Multi Echo (MSME) sequence with TR = 2200 ms and 14 TEs equally spaced from 7 to 90 ms, Matrix size = 96 × 96, FOV = 28 × 28 mm^2^, Slice thickness = 0.7 mm, Averages = 1, Slice number = 16. T2 values were extracted using the system’s inbuilt Image Sequence Analysis (ISA T2vtr) software by defining circular ROIs over each tube and averaged over 3 slices.

### In vivo MRI tracking of CD25 microbeads-labeled Tregs

Animal experiments were approved by University of Kentucky’s institutional animal care and use committee (IACUC #2019-3341) and all experiments were conducted in accordance with IACUC guidelines and reported in accordance with ARRIVE guidelines. Fresh human Tregs (CD25+) cells were incubated for 6 h with 10 µl CD25 microbeads per million Tregs (45 µgFe/ml) and maintained at a concentration of 2 × 10^6^ cells/ml of full media in 6-well plates. After incubation, excess microbeads were removed by three washes followed by centrifugation. Fresh human T cells (CD25-) were prepared under the same conditions. Cell samples were resuspended at a concentration of 1.5 × 10^7^ cells in 100 µl PBS for intravenous injection. Twelve six to eight week-old female NSG mice were sourced from Jackson Laboratory (strain #005557). Liver MRI prescans were acquired for all mice prior to intravenous injections. Five mice received CD25 microbead-labeled Tregs, five mice received CD25 microbead labeled T cells (negative control) and 2 mice received CD25 microbeads alone (20 µl diluted in 80 µl PBS, final concentration of 450 µgFe/ml, positive control). Liver MRIs were acquired again 24 h post-injection.

MRI images were acquired on the same system as above (Bruker BioSpec 7 T) using a mouse birdcage body coil. Axial images of mouse liver were acquired using a Turbo RARE sequence with parameters TR/TE = 2000/20 ms, Matrix size = 128 × 128, FOV = 36 × 24 mm^2^, Slice thickness = 1.2 mm, Averages = 2, Slice number = 16 and a T2map Multi Slice Multi Echo (MSME) sequence with TR = 2000 ms and 10 TEs equally spaced from 6.4 to 64 ms, Matrix size = 128 × 128, FOV = 36 × 24 mm^2^, Slice thickness = 1.2 mm, Averages = 3, Slice number = 16. T2 values were extracted using the system’s inbuilt Image Sequence Analysis (ISA T2vtr, Paravision 360, V3.0) software by defining three circular liver ROIs over 3 slices (9 measurements per mouse per time point).

### Statistical analyses

Statistical comparisons between phantoms and cell samples were performed using a t-test. *P* values less than 0.05 were considered significant. For in vivo experiments, a total of 216 T2 observations from 12 mice were available for analysis. Statistical significance was set at *p* ≤ 0.05p ≤ 0.05 and all tests were two-sided. Missing observations were reported and were excluded on an analysis-by-analysis basis. Paired t-tests were performed for comparisons of prescan and 24-h T2 measurements within treatment group and a one-way ANOVA was performed for comparisons between groups. All analyses were done in R programming language, version 4.1.2 (R Foundation for Statistical Computing, Vienna, Austria). All graphics were produced using the R package ggplot2, version 3.3.5 (Hadley Wickham).

## Data Availability

The datasets generated during and/or analyzed during the current study are available from the corresponding author on reasonable request.

## References

[1] Young IR (1981). Enhancement of relaxation rate with paramagnetic contrast agents in NMR imaging. J. Comput. Tomogr..

[2] Lohrke J (2016). 25 years of contrast-enhanced MRI: Developments, current challenges and future perspectives. Adv. Ther..

[3] Bulte JWM, Daldrup-Link HE (2018). Clinical tracking of cell transfer and cell transplantation: Trials and tribulations. Radiology.

[4] Ibrahim, M. A., Hazhirkarzar, B. & Dublin, A. B. in *StatPearls* (2020). 29494094

[5] Blumfield E, Swenson DW, Iyer RS, Stanescu AL (2019). Gadolinium-based contrast agents - review of recent literature on magnetic resonance imaging signal intensity changes and tissue deposits, with emphasis on pediatric patients. Pediatr. Radiol..

[6] Gale EM (2018). A Manganese-based alternative to gadolinium: Contrast-enhanced MR angiography, excretion, pharmacokinetics, and metabolism. Radiology.

[7] Xiao YD (2016). MRI contrast agents: Classification and application (Review). Int. J. Mol. Med..

[8] Rogosnitzky M, Branch S (2016). Gadolinium-based contrast agent toxicity: A review of known and proposed mechanisms. Biometals.

[9] Brunjes R, Hofmann T (2020). Anthropogenic gadolinium in freshwater and drinking water systems. Water Res..

[10] Pankhurst QA, Connolly J, Jones SK, Dobson J (2003). Applications of magnetic nanoparticles in biomedicine. J. Phys. D: Appl. Phys..

[11] Dahl NV, Kaper RF, Strauss WE, Corvino FA, Zivkovic M (2017). Cost-effectiveness analysis of intravenous ferumoxytol for the treatment of iron deficiency anemia in adult patients with non-dialysis-dependent chronic kidney disease in the USA. Clinicoecon. Outcomes Res..

[12] Kowalczyk M, Banach M, Rysz J (2011). Ferumoxytol: A new era of iron deficiency anemia treatment for patients with chronic kidney disease. J. Nephrol..

[13] Lu M, Cohen MH, Rieves D, Pazdur R (2010). FDA report: Ferumoxytol for intravenous iron therapy in adult patients with chronic kidney disease. Am. J. Hematol..

[14] Ahrens ET, Bulte JW (2013). Tracking immune cells in vivo using magnetic resonance imaging. Nat. Rev. Immunol..

[15] Bulte JW, Kraitchman DL (2004). Iron oxide MR contrast agents for molecular and cellular imaging. NMR Biomed..

[16] Chapelin F, Capitini CM, Ahrens ET (2018). Fluorine-19 MRI for detection and quantification of immune cell therapy for cancer. J. Immunother. Cancer.

[17] Arbab AS (2004). Comparison of transfection agents in forming complexes with ferumoxides, cell labeling efficiency, and cellular viability. Mol. Imaging.

[18] Chapelin F (2019). Tumor formation of adult stem cell transplants in rodent arthritic joints. Mol. Im. Biol..

[19] Miltenyi S, Muller W, Weichel W, Radbruch A (1990). High gradient magnetic cell separation with MACS. Cytometry.

[20] Thorek DL, Tsourkas A (2008). Size, charge and concentration dependent uptake of iron oxide particles by non-phagocytic cells. Biomaterials.

[21] Pasquini MC (2012). Comparative outcomes of donor graft CD34+ selection and immune suppressive therapy as graft-versus-host disease prophylaxis for patients with acute myeloid leukemia in complete remission undergoing HLA-matched sibling allogeneic hematopoietic cell transplantation. J. Clin. Oncol..

[22] Keever-Taylor CA (2012). Characteristics of CliniMACS(R) System CD34-enriched T cell-depleted grafts in a multicenter trial for acute myeloid leukemia-Blood and Marrow Transplant Clinical Trials Network (BMT CTN) protocol 0303. Biol. Blood Marrow Transpl..

[23] Wu YJ (2007). In vivo leukocyte labeling with intravenous ferumoxides/protamine sulfate complex and in vitro characterization for cellular magnetic resonance imaging. Am. J. Physiol. Cell Physiol..

[24] Fink C (2018). (19)F-perfluorocarbon-labeled human peripheral blood mononuclear cells can be detected in vivo using clinical MRI parameters in a therapeutic cell setting. Sci. Rep..

[25] Bulte JW (1992). Specific MR imaging of human lymphocytes by monoclonal antibody-guided dextran-magnetite particles. Magn. Reson. Med..

[26] Yeh TC, Zhang W, Ildstad ST, Ho C (1993). Intracellular labeling of T-cells with superparamagnetic contrast agents. Magn. Reson. Med..

[27] Jin WN (2016). Non-invasive tracking of CD4+ T cells with a paramagnetic and fluorescent nanoparticle in brain ischemia. J. Cereb. Blood Flow Metab..

[28] Liu L (2012). Tracking T-cells in vivo with a new nano-sized MRI contrast agent. Nanomedicine.

[29] Chandrasekharan P (2021). Non-radioactive and sensitive tracking of neutrophils towards inflammation using antibody functionalized magnetic particle imaging tracers. Nanotheranostics.

[30] Gordon-Wylie SW (2020). Measuring protein biomarker concentrations using antibody tagged magnetic nanoparticles. Biomed. Phys. Eng. Express..

[31] Garden OA (2006). A rapid method for labelling CD4+ T cells with ultrasmall paramagnetic iron oxide nanoparticles for magnetic resonance imaging that preserves proliferative, regulatory and migratory behaviour in vitro. J. Immunol. Methods.

[32] Motaln H, Schichor C, Lah TT (2010). Human mesenchymal stem cells and their use in cell-based therapies. Cancer.

[33] Cohan SL, Lucassen EB, Romba MC, Linch SN (2019). Daclizumab: Mechanisms of action, therapeutic efficacy, adverse events and its uncovering the potential role of innate immune system recruitment as a treatment strategy for relapsing multiple sclerosis. Biomedicines..

[34] Grassart A, Dujeancourt A, Lazarow PB, Dautry-Varsat A, Sauvonnet N (2008). Clathrin-independent endocytosis used by the IL-2 receptor is regulated by Rac1, Pak1 and Pak2. EMBO Rep..

[35] Charpentier JC, King PD (2021). Mechanisms and functions of endocytosis in T cells. Cell Commun. Signal..

[36] Bulte JW (1993). Selective MR imaging of labeled human peripheral blood mononuclear cells by liposome mediated incorporation of dextran-magnetite particles. Magn. Reson. Med..

[37] Corot C (2004). Macrophage imaging in central nervous system and in carotid atherosclerotic plaque using ultrasmall superparamagnetic iron oxide in magnetic resonance imaging. Invest. Radiol..

[38] Ahrens ET, Flores R, Xu H, Morel PA (2005). In vivo imaging platform for tracking immunotherapeutic cells. Nat. Biotechnol..

[39] Daldrup-Link HE (2011). MRI of tumor-associated macrophages with clinically applicable iron oxide nanoparticles. Clin. Cancer Res..

[40] Khurana A (2012). Intravenous ferumoxytol allows noninvasive MR imaging monitoring of macrophage migration into stem cell transplants. Radiology.

[41] Khurana A (2017). Visualization of macrophage recruitment in head and neck carcinoma model using fluorine-19 magnetic resonance imaging. Magn. Reson. Med..

[42] Rogers WJ, Basu P (2005). Factors regulating macrophage endocytosis of nanoparticles: Implications for targeted magnetic resonance plaque imaging. Atherosclerosis.

[43] Waiczies H (2011). Perfluorocarbon particle size influences magnetic resonance signal and immunological properties of dendritic cells. PLoS ONE.

[44] Moore A (2002). MRI of insulitis in autoimmune diabetes. Magn. Reson. Med..

[45] Ahrens ET, Young WB, Xu H, Pusateri LK (2011). Rapid quantification of inflammation in tissue samples using perfluorocarbon emulsion and fluorine-19 nuclear magnetic resonance. Biotechniques.

[46] Zanganeh S (2016). Iron oxide nanoparticles inhibit tumour growth by inducing pro-inflammatory macrophage polarization in tumour tissues. Nat. Nanotechnol..

[47] Daldrup-Link HE (2017). Detection of stem cell transplant rejection with ferumoxytol MR imaging: Correlation of MR imaging findings with those at intravital microscopy. Radiology.

[48] Keliher EJ (2011). 89Zr-labeled dextran nanoparticles allow in vivo macrophage imaging. Bioconjug. Chem..

[49] Torrieri G (2020). Dual-peptide functionalized acetalated dextran-based nanoparticles for sequential targeting of macrophages during myocardial infarction. Nanoscale.

[50] Qie Y (2016). Surface modification of nanoparticles enables selective evasion of phagocytic clearance by distinct macrophage phenotypes. Sci. Rep..

[51] Hoehn M (2007). Cell tracking using magnetic resonance imaging. J. Physiol..

[52] Rogers WJ, Meyer CH, Kramer CM (2006). Technology insight: In vivo cell tracking by use of MRI. Nat. Clin. Pract. Cardiovasc. Med..

[53] Janjic JM, Srinivas M, Kadayakkara DK, Ahrens ET (2008). Self-delivering nanoemulsions for dual fluorine-19 MRI and fluorescence detection. J. Am. Chem. Soc..

[54] Srinivas M, Heerschap A, Ahrens ET, Figdor CG, de Vries IJ (2010). (19)F MRI for quantitative in vivo cell tracking. Trends Biotechnol..

[55] Gonzales C (2016). In-vivo detection and tracking of T cells in various organs in a melanoma tumor model by 19f-fluorine MRS/MRI. PLoS ONE.

[56] Chapelin F (2017). Fluorine-19 nuclear magnetic resonance of chimeric antigen receptor T cell biodistribution in murine cancer model. Sci. Rep..

[57] Hingorani DV (2019). Cell penetrating peptide functionalized perfluorocarbon nanoemulsions for targeted cell labeling and enhanced fluorine-19 MRI detection. Magn. Reson. Med..

[58] Sakaguchi S, Yamaguchi T, Nomura T, Ono M (2008). Regulatory T cells and immune tolerance. Cell.

[59] Wood KJ (2011). Regulatory T cells in transplantation. Transp. Proc..

[60] Gedaly R (2019). mTOR inhibitor everolimus in regulatory T cell expansion for clinical application in transplantation. Transplantation.

[61] Terry LV, Oo YH (2020). The next frontier of regulatory T cells: Promising immunotherapy for autoimmune diseases and organ transplantations. Front. Immunol..

[62] Oo YH (2019). Liver homing of clinical grade Tregs after therapeutic infusion in patients with autoimmune hepatitis. JHEP Rep..

[63] Partington KM, Jenkinson EJ, Anderson G (1999). A novel method of cell separation based on dual parameter immunomagnetic cell selection. J. Immunol. Methods.

[64] Ostojić G, Lazić D, Zeljković S (2021). Determination of the iron oxide content in bauxite: Comparing ICP-OES with UV–VIS and volumetric analysis. Chem. Pap..

[65] Bullivant JP (2013). Materials characterization of Feraheme/ferumoxytol and preliminary evaluation of its potential for magnetic fluid hyperthermia. Int. J. Mol. Sci..

[66] Thu MS (2012). Self-assembling nanocomplexes by combining ferumoxytol, heparin and protamine for cell tracking by magnetic resonance imaging. Nat. Med..

[67] Khurana A (2013). Iron administration before stem cell harvest enables MR imaging tracking after transplantation. Radiology.

[68] Khurana A (2013). Ferumoxytol: A new, clinically applicable label for stem-cell tracking in arthritic joints with MRI. Nanomedicine (Lond).

[69] Makela AV, Gaudet JM, Foster PJ (2017). Quantifying tumor associated macrophages in breast cancer: A comparison of iron and fluorine-based MRI cell tracking. Sci. Rep..

[70] Knobloch G (2018). Relaxivity of Ferumoxytol at 1.5 T and 3.0 T. Invest. Radiol..

